# Risk factors for perioperative complications following unilateral biportal endoscopic spine surgery

**DOI:** 10.20452/wiitm.2025.17940

**Published:** 2025-01-03

**Authors:** Jiashen Shao, Zhiwu Zhang, Zihan Fan, Hai Meng, Qi Fei

**Affiliations:** Department of Orthopedics, Beijing Friendship Hospital, Capital Medical University, Beijing, China, China

**Keywords:** lumbar disc herniation, lumbar spinal stenosis, perioperative complications, risk

## Abstract

**INTRODUCTION:**

Unilateral biportal endoscopy (UBE) is a minimally invasive technique that has gradually gained popularity in the field of spine surgery.

**AIM:**

The aim of this study was to identify independent risk factors associated with the occurrence of perioperative complications following UBE surgery through a comprehensive retrospective analysis.

**MATERIALS AND METHODS:**

Consecutive patients who underwent UBE at the Department of Orthopedics of Beijing Friendship Hospital between June 2021 and July 2024 were retrospectively analyzed. Data on demographic characteristics, comorbidities, surgery‑related parameters, and perioperative complications were extracted from medical records, and patients who did and did not develop complications were compared. Potential risk factors for perioperative complications were evaluated using univariable and multivariable logistic regression analyses.

**RESULTS:**

In a cohort of 322 patients, perioperative complications were observed in 20 individuals, yielding an overall incidence rate of 6.8%. Occurrence of perioperative complications was associated with higher body mass index (BMI >28 kg/m2; P <0.001), diabetes mellitus (P <0.001), depression (P <0.001), preoperative analgesia (P = 0.03), American Society of Anesthesiologists classifiation (P <0.001), and longer operative time (>180 minutes; P <0.001). In the multivariable logistic regression analysis, surgery duration longer than 180 minutes (odds ratio [OR], 2.8; 95% CI, 1.5–5.4), depression (OR, 2.5; 95% CI, 1.3–4.7), and BMI greater than 28 kg/m2 (OR, 3.1; 95% CI, 1.7–5.9) were identified as independent risk factors for complications.

**CONCLUSIONS:**

This study demonstrates that UBE surgery is an effective and safe minimally invasive technique for the management of lumbar degenerative diseases, with a relatively low complication rate of 6.8%. Longer operative time, preoperative depression, and a higher BMI were identified as independent risk factors for the occurrence of perioperative complications.

## INTRODUCTION

Endoscopic spine surgery has become a cornerstone in the management of var­ious degenerative spinal diseases.^1^ Among the en­doscopic techniques, unilateral biportal endosco­py (UBE) has emerged as a favored minimally in­vasive approach, as it is associated with reduced tissue disruption and expedited postoperative recovery.^2^^,^^3^^,^^4^^,^^5^ UBE requires only 2 small incisions, thus offering significant technical benefits.^6^ Nev­ertheless, intra­ and postoperative complications remain an important concern for clinicians. Un­derstanding and analyzing the risk factors for these complications is essential to improve sur­gical safety and patient prognosis.

Although extensive research has been con­ ducted on complications and their associat­ed risk factors in traditional open and single­ ­channel endoscopic spinal surgeries, there are few studies specifically examining UBE ­related complications.^1^^,^^7^^,^^8^^,^^9^^,^^10^ Given the growing adoption of UBE in clinical practice, there is a pressing need for a systematic analysis of its complica­tion profile. Such an endeavor would not only aid in reducing complication rates but also provide an evidence ­based framework for improving clin­ical decision ­making.

## AIM

The aim of this study was to identify in­ dependent risk factors associated with the oc­currence of complications during and after UBE surgery through a comprehensive retrospective analysis. By systematically collecting and evalu­ ating clinical data, this study investigated the re­ lationship between patient­specific characteris­ tics, surgical parameters, and perioperative com­ plications. Our findings not only contribute to a deeper understanding of UBE­specific compli­ cations but also offer a scientific foundation for optimizing surgical techniques and perioperative management strategies. Ultimately, the results are expected to offer novel insights into the field of spine surgery and support efforts to enhance the safety and effectiveness of UBE procedures.

## MATERIALS AND METHODS

### Patient selection

Consecutive patients who underwent UBE surgery at the Department of Orthopedics of Bei­jing Friendship Hospital between June 2021 and July 2024 were retrospectively analyzed. The sur­gical approaches included endoscopic discectomy, lateral recess decompression, unilateral laminec­tomy with bilateral decompression (UBE­ULBD), and unilateral biportal endoscopic lumbar inter­ body fusion. All procedures were performed by the same medical team.

The patients met the study inclusion criteria if they were older than 18 years, were diagnosed with a degenerative lumbar spine disease (in­cluding lumbar spinal stenosis, lumbar disc her­niation, and lumbar spondylolisthesis), and un­derwent UBE surgery. Exclusion criteria encom­passed 1) age below 18 years; 2) a history of prior lumbar spine surgery, including lumbar decom­ pression surgery or lumbar interbody fusion sur­gery performed at the same level; and 3) a pres­ence of spinal infections, tumors, or tuberculosis.

### Surgical technique

A patient was placed prone on a spine bed under general anesthesia. Using C­arm fluoroscopy, the surface projections of the mid­ line, intervertebral space level, and pedicles were determined, and the surgical table was adjusted to make the target intervertebral space as perpendic­ular to the floor as possible. Two 2­cm incisions were made, 1.5 cm superior and inferior to the in­ter vertebral space level, respectively, in the medi­al line of the ipsilateral pedicle. The left incision was used as an observation channel and the right one served as a working channel. The deep fas­cia was incised perpendicularly to the skin inci­sion. The bilateral channels were dilated using a graduated dilatation cannula, with both dila­tation cannulas accessing the lamina and inter­ vertebral space and intersecting at the location of the lamina and intervertebral space. The sur­geon held the spinal endoscope in the left hand and the instruments in the right hand. Endoscop­ic lenses and instruments were passed through the channels into the surgical site and manipulat­ed in an aqueous medium. The saline height was adjusted to ensure that it was kept at a distance of approximately 70 to 100 cm from the incision. The soft tissue on the surface of the interver­tebral space was handled using radiofrequency probes (BONSS, Jiangsu, China), leading to gradu­al exposure of the inferior margin of the superior lamina, the base of the spinous processes, the ar­ticular process joints, and the superior margin of the inferior lamina. The bone (including part of the inferior margin of the lamina, the superior margin of the lamina, and the articular process) was partially removed using a grinder, a bone cut­ter, and a lamina biter, and the ipsilateral ligamen­ tum flavum was resected following exposure of the distal end of the ipsilateral ligamentum fla­vum; the decompression range was up to the in­ner wall of the pedicle. Part of the base of the spi­nous process was then removed with a grinding drill. The contralateral ligamentum flavum was de­ compressed using the contralateral pedicle wall as a reference. Finally, complete resection of the bi­ lateral ligamentum flavum was performed. After confirming complete decompression, radiofre­quency probes were used for hemostasis. The in­cision was then closed, and a drain was placed.

### Data collection and analysis 

Patient data were ob­tained from the electronic medical record system of the Beijing Friendship Hospital. The collect­ ed demographic and surgical data included age, sex, occupation, place of residence, body mass index (BMI), weight, height, presence of comor­ bidities, such as hypertension (ie, blood pres­ sure ≥140/90 mm Hg), diabetes mellitus, coro­nary artery disease, osteoporosis, cerebrovascu­ lar disease, or hyperlipidemia, history of smok­ing, history of alcohol consumption, history of surgery, duration of pain, presence of anxiety or depression, history of allergies, duration of surgery, duration of hospitalization, diagnosis, number of fusion levels, American Society of An­esthesiologists (ASA) classification, total blood loss, and occurrence of perioperative complica­tions. Perioperative complications were defined as complications that occurred intraoperative­ly or up to 6 weeks after the surgery.^11^ Two in­vestigators were responsible for data extraction, and the third investigator checked the accuracy of the data. All data were entered into a Micro­ soft Excel spreadsheet (Microsoft Corp., Red­mond, Washington, United States) for consis­tency checking and data cleaning.

### Statistical analysis

Univariable and multivari­able logistic regression analyses were conducted to identify independent risk factors for perioper­ative complications. Stepwise regression was em­ployed prior to the multivariable analysis to se­ lect variables. The variables demonstrating sig­nificant correlations (P <0.1) in the univariable analysis were included in the multivariable mod­ el. The R studio 4.4.0 package (The R Foundation for Statistical Computing, Vienna, Austria) was used for statistical analysis. Continuous variables are presented as mean (SD). Categorical variables are shown as number (percentage). The t test or Mann–Whitney test was used for comparison of continuous variables, while the χ2 test or Fisher exact test was employed to compare categorical variables. All statistical tests were 2­tailed, with a P value of less than 0.05 deemed significant.

### Ethics statement 

The experimental protocol was reviewed and approved by the Ethics Committee of the Beijing Friendship Hospital (2022KY087). The study was conducted in accordance with the experimental protocol and the Declaration of Helsinki, and informed consent was obtained from all participants. Except for routine treat­ment during hospitalization, the patients did not receive any other treatment related to this study and were not exposed to additional risks.

## RESULTS

### General characteristics

A total of 322 patients (160 men, 162 women) at a mean (SD) age of 59.1 (14.3) years (range, 19–75 years) met the inclusion criteria. More than 80% of the study population were older than 40 years. The mean (SD) BMI of the patients was 26 (3.6) kg/m2. De­ tails regarding demographic characteristics and perioperative outcomes are shown in TABLE 1.

### Perioperative complications

In a cohort of 322 patients, perioperative complications were ob­served in 20 cases, yielding an overall incidence rate of 6.8%. The most prevalent complication was dural tear, occurring in 8 patients (2.5%). All instances of dural rupture were managed intra­ operatively through the application of an artifi­cial dural patch. Other notable complications in­cluded wound infection and delayed wound heal­ing (3 patients [0.9%] each). Most of these cas­es responded favorably to antibiotic therapy and routine wound care, except for 1 patient who developed a deep wound infection necessitating in­ travenous antibiotics and surgical debridement. 

Postoperative recurrence of disc herniation was recorded in 3 patients (0.9%). One individ­ual experienced exacerbation of lower extremity pain on the second postoperative day, which was promptly managed with revision surgery, result­ing in symptom resolution. The other 2 patients presented with early postoperative recurrences. One of them underwent open posterior lumbar revision surgery, while the other was successful­ly treated with a conservative approach. Postoperative epidural hematomas were report­ed in 2 patients (0.6%), both of whom exhibited acute worsening of lower extremity symptoms. Diagnosis was confirmed on postoperative com­ puted tomography (CT) or magnetic resonance imaging (MRI). One patient achieved symptom­atic improvement with conservative treatment, whereas the other required surgical evacuation of the hematoma. Additionally, 1 patient devel­oped transient postoperative lower extremity hypomobility, which resolved completely after 3 months of conservative treatment.

### Univariable and multivariable analysis

Univariable analysis showed that factors such as diabetes, lon­ ger surgery duration (>180 min), depression, pre­ operative analgesia, ASA classification, and BMI were significantly associated with perioperative complications after UBE surgery TABLE 1.

In multivariable logistic regression analy­ sis, surgery duration longer than 180 minutes (odds ratio [OR], 2.8; 95% CI, 1.5–5.4), depres­sion (OR, 2.5; 95% CI, 1.3–4.7), and BMI great­er than 28 kg/m2 (OR, 3.1; 95% CI, 1.7–5.9) were identified as independent risk factors for compli­ cations. These variables remained significantly associated with complications even after adjust­ment for other factors TABLE 2.

## DISCUSSION 

UBE has emerged as a promising minimally invasive technique in spinal surgery, offering benefits such as reduced tissue trauma and faster recovery times.^12^ The results of a re­cent meta­analysis of 528 patients undergoing UBE surgery showed significant improvement in both Visual Analog Scale and Oswestry Disabil­ity Index scores after UBE surgery, as compared with preoperative results. These findings demon­strate that UBE is associated with favorable clin­ical outcomes.^13^ However, similarly to all surgi­cal procedures, UBE is not without risks, partic­ularly in terms of perioperative complications. The incidence of such complications, including infections, nerve injuries, and dural rupture, re­ mains a critical concern for clinicians.^14^ Despite growing adoption of UBE, there are few compre­hensive studies investigating perioperative com­ plications specifically associated with this tech­nique, as well as the factors that may predispose patients to these adverse outcomes. Identifying these factors is crucial not only for improving sur­gical outcomes but also for developing targeted strategies to mitigate risks. In this study, we not­ ed an overall complication rate of 6.8%, and iden­tified 3 factors associated with an increased risk of complications, including operative time great­ er than 180 minutes, depression, and BMI great­ er than 28 kg/m2.

Prolonged operative time has been consistently associated with an increased risk of perioperative complications in all types of surgery.^15^^,^^16^^,^^17^ For UBE surgeries, an operative time longer than 180 min­utes may result in a higher incidence of compli­cations, such as infection, increased blood loss, and delayed wound healing.^18^ There are several mechanisms behind effect. First, prolonged sur­gical time increases the duration of tissue expo­ sure to the external environment, thus enhancing the risk of infection. In addition, prolonged sur­gery may lead to more bleeding and tissue dam­ age, as well as extended postoperative recovery time, all of which may affect the complication rates. Second, prolonged anesthesia places an additional burden on the patient’s physiological function and may cause anesthesia ­related com­plications, such as cardiovascular and respiratory problems. It also increases the risk of postoper­ative cognitive dysfunction, especially in elderly patients.^19^ Third, prolonged surgical times often reflect the complexity and difficulty of the proce­dure, and complex surgeries are inherently more prone to complications. Long and demanding procedures are also a great test of the physical strength and concentration of the surgical team, and may increase the risk for operational errors and complications. All things considered, short­ ening the duration of surgery as much as possible by optimizing the surgical process and improv­ ing surgical efficiency could significantly reduce the incidence of postoperative complications in spine surgery and improve the quality of patient postoperative recovery.

**TABLE 1:** Characteristics of patients with and without complications following unilateral biportal endoscopic spine surgery

Parameter		Patients without complications (n = 302)	Patients with complications (n = 20)	*P* value
Age, y, mean (SD)		58.92 (14.3)	63 (9.7)	0.36
Age group, y	18–39	48 (15.9)	0	–
	40–59	62 (20.5)	6 (30)	
	≥60	192 (63.6)	14 (70)	
Sex	Men	152 (50.3)	8 (40)	0.53
	Women	150 (49.7)	12 (60)	
Occupation (manual work)		189 (62.6)	13 (65)	>0.99
Area of residence (rural)		106 (35.1)	8 (40)	0.84
Educational attainment (≥12 years)		119 (39.4)	9 (45)	0.6
BMI, kg/m2, mean (SD)		25.88 (6.1)	28.97 (10.5)	0.01
BMI >28 kg/m2		32 (10.6)	12 (60)	<0.001
Hypertension		142 (47)	12 (60)	0.43
Diabetes		40 (13.2)	12 (60)	<0.001
Coronary artery disease		14 (4.6)	2 (10)	0.46
Current smoking		50 (16.6)	2 (10)	0.59
Alcohol consumption		30 (9.9)	0	0.99
Osteoporosis		118 (39.1)	8 (40)	0.95
Cerebrovascular disease		24 (7.9)	4 (20)	0.21
Hyperlipidemia		50 (16.6)	6 (30)	0.29
History of surgery		178 (58.9)	16 (80)	0.2
Chronic pain (>3 years)		116 (38.4)	14 (70)	0.06
Anxiety		78 (25.8)	6 (30)	0.77
Depression		48 (15.9)	16 (80)	<0.001
Preoperative analgesia		56 (18.5)	10 (50)	0.03
ASA classification	I	136 (45)	4 (20)	<0.001
	II	140 (46.4)	8 (40)	
	III–IV	26 (8.6)	8 (40)	
Diagnosis	Radiculopathy with IVD	180 (59.6)	12 (60)	0.87
	Spinal stenosis	118 (39.1)	8 (40)	
	Cauda equina	4 (1.3)	0	
Operated segment	L1–L2	2 (0.7)	0	0.44
	L2–L3	4 (1.3)	0	
	L3–L4	24 (7.9)	0	
	L4–L5	182 (60.3)	10 (50)	
	L5–S1	56 (18.5)	6 (30)	
	Multilevel	34 (11.3)	4 (20)	
Type of surgery	Discectomy	170 (56.3)	8 (40)	0.33
	Lateral recess decompression	76 (25.2)	8 (40)	
	ULBD	46 (15.2)	4 (20)	
	Other	10 (3.3)	0	
Total blood loss, ml, mean (SD)		56.77 (20.6)	68.50 (28.7)	0.67
Surgical duration, min, mean (SD)		125.45 (30.1)	146 (25.6)	0.32
Surgical duration ≥180 min		38 (12.6)	14 (70)	<0.001
Total hospitalization costs, RMB, mean (SD)		47 573.37 (300.5)	48 507.43 (250.4)	0.66
Length of hospitalization, d, mean (SD)		9.2 (2.2)	12 (4.5)	0.17

Data are presented as number (percentage) unless indicated otherwise.

P <0.05 was considered significant.

Abbreviations: ASA, American Society of Anesthesiologists; BMI, body mass index; IVD, intervertebral disc degeneration; RMB, Chinese yuan renminbi; ULBD, unilateral laminectomy with bilateral decompression

**TABLE 2:** Multivariable analysis of factors independently associated with complications following unilateral biportal endoscopic surgery

Variable		OR	95% CI	*P* value
Diabetes	5.48	0.43–70.52	0.19	
Surgical duration ≥180 min	16.61	1.7–162.42	0.02	
Depression		9.32	1.11–78.64	0.04
Preoperative analgesia		2.65	0.29–24.57	0.39
ASA classification	I	Reference	–	–
	II	0.65	0.04–9.51	0.75
	III–IV	3.53	0.23–54.59	0.37
BMI >28 kg/m2		16.13	1.62–160.24	0.02

Abbreviations: OR, odds ratio; others, see TABLE 1

Psychiatric disorders, particularly depression, have been identified as important risk factors for postoperative complications. Chronic pain is of­ ten accompanied by a depressive state. Depres­sion significantly influences the development of postoperative complications in spine surgery, and its mechanisms of action are complex and mul­tifaceted. Biologically, patients with depression often present with abnormal immune function, including decreased lymphocyte activity and ele­vated levels of proinflammatory cytokines, leading to poor wound healing and increased risk of infection. In addition, depression is associated with dysregulation of the hypothalamic­pituitary­ ­adrenal axis, causing elevation of cortisol levels, which further impairs immune response and tis­sue repair.^20^ On a psychological level, depressed patients are more sensitive to pain perception, which not only makes postoperative pain man­agement more difficult but may also lead to over­ use of analgesic medications (especially opioids), increasing the risk of complications such as re­ spiratory depression and gastrointestinal prob­lems. Behaviorally, depression often leads to poor adherence to postoperative care, including de­ creased willingness to take medications on sched­ ule, attend regular follow ­up appointments, and participate in physical therapy, all of which in­ crease the risk of poor wound healing and unde­tected complications.^21^ In addition, depression is often associated with poor lifestyle habits, such as smoking, inadequate diet, and lack of exer­cise, which further impact postoperative recovery. Therefore, comprehensive preoperative assess­ ment and multidisciplinary collaborative manage­ment of depression, combined with psychological support and optimized rehabilitation programs, are essential to reduce the incidence of perioper­ative complications and improve the prognosis of patients undergoing spine surgery.^22^ In patients scheduled for UBE surgery, mental health status should be carefully assessed preoperatively. In­terventions such as preoperative counseling, op­timized antidepressant treatment, and postop­erative psychological support may help reduce the risks and improve overall surgical outcomes.

BMI greater than 28 kg/m2 was shown to have a significant impact on the development of peri­ operative complications in spine surgery. This association involves several mechanisms. First, high BMI is closely associated with greater intra­ operative technical difficulty. Excess adipose tis­ sue restricts the surgical field of view and reduc­es the operating space, which makes the surgical procedure more complex and elevates the risk of intraoperative blood loss and postoperative in­ fection.^23^^,^^24^ In addition, high BMI affects anes­thesia management and increases the likelihood of anesthesia­related complications, such as dif­ficult airway management and postoperative re­spiratory depression. During the postoperative recovery phase, patients with obesity tend to ex­perience limited mobility and greater difficulty in rehabilitation, which may further prolong hospi­talization and postoperative recovery time.^25^ To mitigate these risks, comprehensive preoperative assessment, individualized surgical and anesthet­ic plans, and specialist postoperative care and re­ habilitation support should be provided to opti­mize surgical outcomes and quality of postoper­ative recovery in patients with high BMI.

This study has several limitations that should be considered when interpreting the findings. First, its retrospective design makes it inherent­ly subject to a selection bias and incomplete data collection, potentially impacting the accuracy and reliability of the results. Second, the relatively small sample size, as compared with multicenter retrospective studies, may limit the statistical power, particularly in the analysis of rare com­ plications. However, it appears to be a common limitation of many single­center studies explor­ing endoscopic spinal surgery. The main reason for that is the relatively low complication rate of endoscopic spinal procedures such as UBE, as compared with conventional open surgery. Fur­thermore, as this was a single­center study, there may be considerable variability in surgical tech­niques, postoperative management, and patient selection criteria in comparison with other insti­tutions, which affects generalizability of the out­ comes. Consequently, future research should in­clude larger, multicenter cohorts to validate these findings and enhance their applicability across di­ verse clinical settings.

CONCLUSIONS

This study demonstrated that UBE surgery is an effective and safe minimally invasive technique for the management of lum­ bar degenerative diseases, with a relatively low complication rate of 6.8%. Additionally, we iden­tified operative time greater than 180 minutes, preoperative depression, and a BMI greater than 28 kg/m2 as independent risk factors for the oc­currence of perioperative complications. These findings suggest that careful patient selection and optimization of these risk factors are cru­ cial for minimizing complication rates and im­ proving surgical outcomes in patients undergo­ ing UBE procedures.
